# Progress in Surgery

**Published:** 1899-07-22

**Authors:** 


### Progress in Surgery.

### HERNIA.

(Continued from page 265.)

The third variety, the properitoneal hernia, is rarely recognised, because there is no external or ventral swelling formed by the secondary sac. There is usually a sac in the scrotum or labium. The reduction of hernial contents from the external part of the sac to the other may give rise to the production of *reduction en masse*. In rare cases a femoral sac has had a properitoneal diverticulum. The treatment in reducible cases of interstitial hernia is either by operation or by trusses. The truss should have a special plate extending upwards and outwards. It is recommended in operating to place the partially descended testicle in the subserous tissue in children, and in young adults to remove it if very small. Transplantation is not advised in any radical cures.

A case of prevesical hernia is reported by C. H. Makins.[8] The patient had also a reducibly inguinal hernia on the same side but with a sac quite independent of the other. The writer draws a distinction between "true prevesical hernia" and properitoneal hernia. In the former the sac is independent of any pre-existent or subsequent inguinal hernia; in the latter the sac forms an integral part of the inguinal hernia. He considered that in his case a hernial pouch formed between the epigastric and obliterated hypogastric arteries, and was diverted into the prevesical space by the resistance of the conjoined tendon. Macready thought the hernia was into a congenital crypt of the peritoneum.

**Diagnosis of Hernia.**—F. Eve[9] relates some cases in young children in which there was difficulty in diagnosis between strangulated hernia and inflamed undescended testicle and torsion of the testicle, and between hernia irreducible or otherwise and hydrocele of the cord. In one case, a boy of three, with undescended testicle, there was an irreducible swelling in the groin which had existed four days. The bowels acted during the first day, but not after, and there was vomiting, but only with coughing (whooping). The boy seemed bright and well until the night of operation. A gangrenous knuckle of bowel was found. In another case, in a boy of three and a half, there was an irreducible swelling in the groin, which was red and tender. There were no symptoms of strangulation. The testicle was undescended. It was operated upon with the idea that the swelling might be either an inflamed testicle or an incarcerated hernia. The testicle was found to be black, and hanging like a grape from its stalk, and gangrenous from torsion of the cord.

If hydrocele of the cord is irreducible it may closely resemble a hernia. Usually, however, the fingers can be inserted above the hydrocele, and feel the cord free. Also a hydrocele is pulled down by traction upon the testis, whereas a hernia is not. A case is described of hernia complicated with hydrocele of the cord; another of hernia together with hydrocele of the cord and hydrocele of the tunica vaginalis; and a third case in which the testicle occupied the upper part of the swelling, the lowest part being a closed cyst. Probably in the last case the cyst was developed in the process of peritoneum which normally descends into the scrotum in advance of the testicle.

**Accidental Hernia.**—When is a hernia the result of an accident, and when therefore can it be said that a workman is entitled to compensation for its development? C. Kauffmann[9] has recently discussed this question. He says: (1) There must be a history of special strain, not merely of ordinary work, as a cause; (2) there must be, further, a history of the sudden appearance of the hernia, with great pain in the neighbourhood of the rings, compelling the patient to desist from work; (3) clinical examination should be made soon after the hernia appears. In a large proportion the hernia is incarcerated. It must not be larger than a hen's egg, and be either interstitial or project only partially through the external ring. It must not return spontaneously, but require taxis. There should be no hernia on the other side, and the canal should barely admit the finger. Advanced age in a man who has done heavy work for years points against accidental causation.

[8] Lancet, April, 1899. [9] Brit. Med. Jour., Feb., 1899.

